# Supplementary material for: Circulating inflammatory biomarkers and academic performance in adolescents: DADOS study
Source: PLoS One. 2020 Nov 6;15(11):e0242016. doi: 10.1371/journal.pone.0242016 (PMC7647075; doi:10.1371/journal.pone.0242016)
Supplement: S1 Table — (DOCX) [file pone.0242016.s001.docx]

| **S1 Table. Parameters of the receiver operating characteristic curve analysis for the diagnostic performance of circulating inflammatory biomarkers in identifying low academic abilities.** | | | | | |
| --- | --- | --- | --- | --- | --- |
| Low academic abilities |  | White blood cells | Interleukin-6 | Tumor necrosis factor-α | C-reactive protein |
| Verbal | AUC | 0.532 | 0.438 | 0.464 | 0.533 |
|  | 95%CI | 0.459 – 0.606 | 0.358 – 0.518 | 0.391 – 0.538 | 0.459 – 0.606 |
|  | p | 0.385 | 0.129 | 0.337 | 0.382 |
|  | Cut-off | - | - | - | - |
|  | Sensitivity (%) | - | - | - | - |
|  | Specificity (%) | - | - | - | - |
| Numeric | AUC | 0.482 | 0.477 | 0.497 | 0.433 |
|  | 95%CI | 0.407 – 0.557 | 0.398 – 0.557 | 0.423 – 0.571 | 0.359 – 0.508 |
|  | p | 0.636 | 0.582 | 0.939 | 0.078 |
|  | Cut-off | - | - | - | - |
|  | Sensitivity (%) | - | - | - | - |
|  | Specificity (%) | - | - | - | - |
| Reasoning | AUC | 0.514 | 0.475 | 0.535 | 0.526 |
|  | 95%CI | 0.441 – 0.586 | 0.396 – 0.554 | 0.462 – 0.608 | 0.453 – 0.599 |
|  | p | 0.713 | 0.536 | 0.346 | 0.486 |
|  | Cut-off | - | - | - | - |
|  | Sensitivity (%) | - | - | - | - |
|  | Specificity (%) | - | - | - | - |
| Overall | AUC | 0.547 | 0.466 | 0.529 | 0.531 |
|  | 95%CI | 0.475 – 0.619 | 0.387 – 0.545 | 0.456 – 0.601 | 0.459 – 0.604 |
|  | p | 0.203 | 0.404 | 0.436 | 0.398 |
|  | Cut-off | - | - | - | - |
|  | Sensitivity (%) | - | - | - | - |
|  | Specificity (%) | - | - | - | - |
| AUC: area under the curve; CI: confidence interval. | | | | | |
