# Supplementary material for: Circulating inflammatory biomarkers and academic performance in adolescents: DADOS study
Source: PLoS One. 2020 Nov 6;15(11):e0242016. doi: 10.1371/journal.pone.0242016 (PMC7647075; doi:10.1371/journal.pone.0242016)
Supplement: Attachment — Submitted filename: Response to Reviewers.docx [file pone.0242016.s002.docx]

**Responses to the reviewers**

**Manuscript No. PONE-D-20-17331**

Dear Samson Gebremedhin,

My co-authors and I are pleased to respond the editor’s and the reviewers’ comments and resubmit a revised version of the paper entitled: “Circulating inflammatory biomarkers and academic performance in adolescents: DADOS study” submitted as a Research article to PLOS ONE (No. PONE-D-20-17331). Changes to the original manuscript appear in blue text for ease of identification, and we believe that our manuscript is stronger as result of these modifications. An itemized point-by-point response to the journal requirements and the reviewers’ comments is presented below.

Yours sincerely,

The authors

**Editor Comments:**

**Comment 1**

What was the basis for considering sex, pubertal stage, parental educational level, type of school, waist circumference and adherence to the Mediterranean diet were included as covariates? Is there any evidence that variables like waist circumference and adherence to the Mediterranean diet affect the outcome of interest?

**Response 1**

Thank you for the question. This issue was deeply discussed among authors, and finally, we decided to include sex, pubertal stage, parental educational level, type of school, waist circumference, and adherence to the Mediterranean diet as covariates due to their influence on the study variables (inflammation and academic performance). Regarding the editor’s question about waist circumference, prior research in adolescents has shown that having abdominal obesity or high adiposity was associated with lower academic performance (individually or in combination with unhealthy dietary habits) [1]. Likewise, previous studies have suggested that adherence to the Mediterranean diet may be positively associated with academic performance in adolescents [2, 3]. Thus, following the editor’s comment, this information has been included in the manuscript. Please, see lines 139 to 145, and 326 to 330.

1. Correa-Burrows P, Rodriguez Y, Blanco E, Gahagan S, Burrows R. Increased adiposity as a potential risk factor for lower academic performance: A cross-sectional study in Chilean adolescents from low-to-middle socioeconomic background. Nutrients. 2018;10(9):1133.
2. Adelantado-Renau M, Beltran-Valls MR, Esteban-Cornejo I, Martínez-Vizcaíno V, Santaliestra-Pasías AM, Moliner-Urdiales D. The influence of adherence to the Mediterranean diet on academic performance is mediated by sleep quality in adolescents. Acta Paediatr. 2019;108(2):339-346.
3. Vassiloudis I, Yiannakouris N, Panagiotakos DB, Apostolopoulos K, Costarelli V. Adherence to the Mediterranean diet and specific lifestyle habits are associated with academic performance in Greek adolescents. Med J Nutrition Metab. 2017;10(2):93-103.

**Comment 2**

In the study economic status related variables have not been measured and adjusted. At least theoretically, low household economic may lead to both of inflammation and low academic performance and confound the relationship of interest. Why it was not possible to measure and adjust such variables in the study?

**Response 2**

Thank you for the question. Authors agree with the editor that socioeconomic status may play a key role in both, inflammatory state and academic performance. Thus, in the present study, several socioeconomic status related variables were included as covariates. Specifically, parental educational level, type of school, waist circumference and adherence to the Mediterranean diet, which are related to socioeconomic status, were included as covariates. Regarding parental education level and type of school, both are proxies of socioeconomic status. Indeed, adolescents’ socioeconomic status generally refers to the amount of parents' income and level of education, which is also reflected in the type of school [1]. In addition, waist circumference and adherence to the Mediterranean diet have been also associated with socioeconomic status [2,3]. Thus, the inclusion of socioeconomic related variables in the current study has been clarified in the manuscript. Please, see lines 139 to 144.

1. Vukojević M, Zovko A, Talić I, Tanović M, Rešić B, Vrdoljak I, et al. Parental socioeconomic status as a predictor of physical and mental health outcomes in children - Literature review. Acta Clin Croat. 2017;56(4):742–8.
2. Newton S, Braithwaite D, Akinyemiju TF. Socio-economic status over the life course and obesity: Systematic review and meta-analysis. PLoS ONE. 2017;12(5):e0177151.
3. Bonaccio M, Bonanni A, Donati MB, de Gaetano G, Iacoviello L. Chapter 43 - Socioeconomic determinants of the adherence to the Mediterranean diet. In: Preedy VR, Watson RR, editors. The Mediterranean Diet. An Evidence-based Approach. 2nd ed. New York: Elsevier, Academic Press; 2020. pp. 495-501.

**Comment 3**

Please state the statistical basis for reaching at the sample size of 244.

**Response 3**

Thank you. Although in this manuscript we present cross-sectional data, our research project was designed as a 3-year longitudinal study aimed to analyse the longitudinal associations of physical activity with psychological wellness, fitness and cognition in adolescents (athletes and non-athletes). Thus, the sample size calculation was performed according to the longitudinal design of the study taking into consideration the 50^th^ percentile of the Body Mass Index (BMI; 21±3 kg/m^2^) in adolescents between 14 and 16 years old [1]. Our original hypothesis was that adolescents who practised competitive physical activity (athletes) would present a BMI reduction during adolescence of 1.5 kg/m^2^ compared to the reference population (non-athletes), that is a value of 19.5 kg/m^2^. Based on power calculation for detecting a medium sized effect with 80% power and with a level of significance of 0.05, and taking into account a loss of 20% each year, the sample size was of 150 adolescents in each group (athletes and non-athletes), with a total sample of 300 adolescents. However, from the total DADOS study sample (n = 274), in the present cross-sectional study, adolescents only were included if they had valid data for at least one circulating inflammatory biomarker and academic performance, reaching at the sample size of 244. Thus, following the editor’s suggestion, brief information regarding the sampling process has been included in the manuscript. Please, see lines 84 to 90.

1. Moreno LA, Mesana MI, González-Gross M, Gil CM, Fleta J, Wärnberg J, et al. Anthropometric body fat composition reference values in Spanish adolescents. The AVENA Study. Eur J Clin Nutr. 2006;60(2):191.6.

**Comment 4**

Results: I don’t see any sub-section that summarizes the findings of the logistic regression analysis. Please include a short paragraph or so.

**Response 4**

Thank you for the suggestion. Results of the logistic regression analyses were at the end of the results section (please, see lines 239 to 246). Thus, following the editor’s comment and with the intention to make it easier to find the different results, several sub-sections have been included throughout the results section. Please, see lines 210, 217, 227 to 228, and 237 to 238.

**Comment 5**

Discussion: Please discuss limitation of the study including residual confounding from unmeasured variables and possible inflation of the type I error rate due to multiple testing.

**Response 5**

Thank you for this important suggestion. Following the editor’s comment, this issue has been discussed in the limitation section. Please, see lines 317 to 324.

**Comment 6**

It’s good that only students free from chronic diseases were included in the study. But it is not clear how the health status of the students was assessed.

**Response 6**

Thank you. Following the editor’s comment, information about the assessment of participants’ health status has been included in the manuscript. Please, see lines 79 to 82.

Additional Editor Comments:

**Comment 7**

Table 1: Some of the significant differences between the two genders are not identified (e.g. academic scores for English and Spanish).

**Response 7**

Thank you very much for the comment. Significant differences between the two genders have been highlighted in bold. Please, see Table 1.

**Journal Requirements:**

When submitting your revision, we need you to address these additional requirements.

**Requirement 1.** Please ensure that your manuscript meets PLOS ONE's style requirements, including those for file naming. The PLOS ONE style templates can be found at

https://journals.plos.org/plosone/s/file?id=wjVg/PLOSOne_formatting_sample_main_body.pdf and

https://journals.plos.org/plosone/s/file?id=ba62/PLOSOne_formatting_sample_title_authors_affiliations.pdf

**Response:**

Authors have carefully revised PLOS ONE's style requirements, and the manuscript has been adapted. Nevertheless, if some issue is missing or wrong, please, let us know and we will be pleased to make the appropriate modifications. Thank you.

**Requirement 2.** Please ensure that you include a title page within your main document. We do appreciate that you have a title page document uploaded as a separate file, however, as per our author guidelines (http://journals.plos.org/plosone/s/submission-guidelines#loc-title-page) we do require this to be part of the manuscript file itself and not uploaded separately.

Could you therefore please include the title page into the beginning of your manuscript file itself, listing all authors and affiliations?

**Response:**

Thank you. Following the author guidelines, the title page, listing all authors and affiliations, has been included at the beginning of the manuscript. Please, see page 1.

**Requirement 3.** Thank you for stating the following in the Financial Disclosure section:

"The DADOS Study is funded by the Spanish Ministry of Economy and Competitiveness, MINECO (DEP2013-45515-R) and by the Jaume I University of Castellon, UJI (P1·1A2015-05; UJI-A2019-12).

This work is partly supported by a Sunny Sport research grant from the Schweppes Suntory Spain Company. MAR was supported by grant UJI E-2018-21. J.M was supported by grants FCT SFRH/BSAB/142983/2018 and UID/DTP/00617/2019, as well as Programa de Bolsas Santander Universidades 2018."

We note that you received funding from a commercial source: Schweppes Suntory Spain Company.

Please provide an amended Competing Interests Statement that explicitly states this commercial funder, along with any other relevant declarations relating to employment, consultancy, patents, products in development, marketed products, etc.

Within this Competing Interests Statement, please confirm that this does not alter your adherence to all PLOS ONE policies on sharing data and materials by including the following statement: "This does not alter our adherence to PLOS ONE policies on sharing data and materials.” (as detailed online in our guide for authors http://journals.plos.org/plosone/s/competing-interests). If there are restrictions on sharing of data and/or materials, please state these. Please note that we cannot proceed with consideration of your article until this information has been declared.

Please include your amended Competing Interests Statement within your cover letter. We will change the online submission form on your behalf.

Please know it is PLOS ONE policy for corresponding authors to declare, on behalf of all authors, all potential competing interests for the purposes of transparency. PLOS defines a competing interest as anything that interferes with, or could reasonably be perceived as interfering with, the full and objective presentation, peer review, editorial decision-making, or publication of research or non-research articles submitted to one of the journals. Competing interests can be financial or non-financial, professional, or personal. Competing interests can arise in relationship to an organization or another person. Please follow this link to our website for more details on competing interests: http://journals.plos.org/plosone/s/competing-interests

**Response:**

A competing interests statement about the role of Schweppes Suntory Spain Company in our study has been included in the cover letter. Please, see the cover letter.

**Requirement 4.** We note that you have included the phrase “data not shown” in your manuscript. Unfortunately, this does not meet our data sharing requirements. PLOS does not permit references to inaccessible data. We require that authors provide all relevant data within the paper, Supporting Information files, or in an acceptable, public repository. Please add a citation to support this phrase or upload the data that corresponds with these findings to a stable repository (such as Figshare or Dryad) and provide and URLs, DOIs, or accession numbers that may be used to access these data. Or, if the data are not a core part of the research being presented in your study, we ask that you remove the phrase that refers to these data.

**Response:**

Thank you for the comment. Following PLOS ONE's data sharing requirements, the phrase “data not shown” has been removed, and data are presented in S1 Table. The following citation “(S1 Table)” has been included in the manuscript (please, see lines 234 to 235), and a supporting information file is attached in the submission system. Please see “Supporting information file_S1 Table”.

**Reviewer #1:**

**Comment 1**

The authors present a cross-sectional study of 244 boys and girls in order to test the hypothesis that inflammation mediators are associated with lower academic achievements.

The background information correctly argues for the feasibility of the hypothesis, and cite correct and up-to-date references.

**Response 1**

We would like to thank the reviewer for giving us the opportunity to revise our manuscript.

**Comment 2**

Children were selected from both public and private schools, and all were on the same grade. The authors may wish to comment how many schools were approached and their possible representativeness in their community.

**Response 2**

We thank you very much for the comment. Participants of the present study came from 38 different high schools, being 50% of them private schools. This information and its representativeness have been included in the “Study design and sample selection” section. Please, see lines 87 to 89.

**Comment 3**

Little is shown about the actual recruiting process. The authors state the children were healthy and without chronic disease, they may wish to describe how this was evaluated. Furthermore, these mediators are also related to acute conditions; such as respiratory, gastrointestinal or dental problems, which may be more frequent in this age group. Some acute ailments may show seasonal variations during the year and it is possible to have several children affected at once. The authors may wish to comment on this.

**Response 3**

Thank you very much for the comments. Following the reviewer’s comments, additional information about the recruiting process has been included in the manuscript. Moreover, authors have clarified what “free of any chronic disease” means and stated how adolescents’ health status was assessed. Please, see lines 76 to 84. Finally, authors agree with the reviewer that there are important factors which may influence inflammation in adolescents. Regarding seasonal related variables, it is likely that these factors did not have a great influence on the study associations, since data were collected during the same season of the year (i.e., from March to May, 2015), as we have stated in the methods section (please, see lines 75 to 76). However, the reviewer highlights a very relevant aspect when referring to acute conditions, which were self-reported in the present study. Thus, although participants were apparently healthy, the absence of a physician diagnosis could influence our results. Therefore, this information has been mentioned in the manuscript, and included as a limitation of the study. Please, see lines 90 to 93, and 317 to 323.

**Comment 4**

To my knowledge inflammation mediators (IL-6 and TNF) were correctly assessed, CRP and leukocytes were also correctly measured (though, these are not mediators, but rather may show the effect of the mediators). Academic performance was measured by both, grade point average on several subject maters and on a standarized test. It is important that the authors state if this test was applied at the same time of the measurement of mediators.

**Response 4**

Authors thank you very much for the appreciation. Both inflammatory biomarkers and academic abilities were evaluated within the same week, but not always the same day. Thus, since this is an important issue to consider, this information has been included in the manuscript. Please, see lines 124 to 127.

**Comment 5**

Covariates for the analysis were sex, pubertal development, parental educational achievement, type of school, and compliance with Mediterranean diet. Body mass index and waist circumference were also considered.

**Response 5**

Following an editor’s suggestion, additional information about the rationality for including these covariates in the analyses has been included in the manuscript. Please, see lines 139 to 145.

**Comment 6**

Logistic regression was performed with the value that best discriminated in ROC curve analysis; between achievement and the mediators. Since the values obtained with ROC curve analysis are close to the median of achievement (between 5 and 6); it would be important to know how these ratings relate to fail-pass grades in the Spanish educational system.

**Response 6**

The values obtained from the receiver operating characteristic (ROC) curves analyses represent the cut-off points at which inflammatory biomarkers have the ability of discriminating low academic performance. In our study, white blood cells showed discriminatory ability to identify low academic performance in English with a cut-off point of 5.25 10^3^/μL. Similarly, tumor necrosis factor- α showed discriminatory ability to identify low academic performance in all the academic grades, with a concentration ranging from 5.75 to 5.85 pg/mL. Thus, authors agree with the reviewer that, using these cut-off points and logistic regression analysis, it would be interesting to analyse the association between high circulating inflammatory biomarkers (i.e., above the cut-off values provided by the ROC curve analysis) and the likelihood of repeating an academic grade. In the Spanish educational system, in secondary education, Spanish students should repeat the academic year whether they have failed more than two subjects, or if they have failed these specific two subjects: Maths and Language. In this sense, although we think that the suggested analyses are interesting, our research project does not have enough statistical power to conduct them, since only 7 adolescents (2.9%) repeated the academic grade in our sample. Please, see Table below.

**Comment 7**

In the end TNF was associated to most of academic achievement ratings, and CRP was associated with verbal abilities ratings. In the discussion, the authors explain their findings with previously published possible effects of both TNF and CRP in the central nervous system. Yet, the reasons for having higher mediators values are not fully explained. I believe that Mediterranean diet may not explain the full extent of these abnormalities. The authors may wish to comment on the degree of abnormality (i.e. compare these differences to normal values in their laboratory).

**Response 7**

Thank you. Following the reviewer’s comment, the discussion section has been extended, including other possible reasons to explain our results. Please, see lines 277 to 287. In addition, as suggested by the reviewer, the speculation related to inflammatory levels has been included in the manuscript. Please, see lines 300 to 303.

**Comment 8**

The greatest confounder in these circumstances tends to be obesity. ¿how many of their children were obese or overweight?, even if they used waist circumference in their logistic analysis.

**Response 8**

Thank you for the question. Waist circumference is a central fat measure related to cardiometabolic diseases which has shown a great relationship with inflammation [1] and academic performance [2]. This last study indicated that waist circumference could be more clearly related to academic performance than obesity. In addition, in our sample, only 12% of adolescents present overweight or obesity. Thus, after a deep discussion, authors thought that could be plausible that waist circumference had a greater influence on our results than obesity, and decided to include waist circumference as a covariate. Nevertheless, we agree with the reviewer’s comment that it would be interesting to include descriptive data regarding overweight and obesity. Therefore, following the reviewer’s comment, the percentage of adolescents with overweight/obesity has been included in Table 1. Moreover, information regarding how this issue was evaluated has been added in the methods section. Please, see lines 163 to 165.

1. Al-Domi H, Al-Shorman A. Increased waist circumference is associated with subclinical atherosclerosis in schoolchildren. Diabetes Metab Syndr Clin Res Rev. 2019;13(1):264–9.
2. Correa-Burrows P, Rodriguez Y, Blanco E, Gahagan S, Burrows R. Increased adiposity as a potential risk factor for lower academic performance: A cross-sectional study in Chilean adolescents from low-to-middle socioeconomic background. Nutrients. 2018;10(9):1133.

**Comment 9**

The association makes perfect sense. Beyond the possible effects of TNF on the brain, If a child doesn’t feel well his/her performance will be substandard. I believe the authors should comment on this aspects before the paper can be published.

**Response 9**

Thank you very much for the suggestion. We agree with the reviewer that adolescents’ health status could influence the study variables. However, participants self-reported whether they had experienced symptoms of any general health problem (e.g., cold, infection, fever, allergies) or dental problems (e.g., toothache) during the past week, as well as at the time of testing. Thus, participants who reported some of these health problems were given an appointment some weeks later when they self-reported not to be experiencing health problems, and we assumed that adolescents recruited for the present study were apparently healthy. Since this is an important point, this information has been included in the methods section. Please, see lines 90 to 93. However, since acute conditions were not assessed by a physician, this issue has been included as a limitation of the study. Please, see lines 317 to 322.

**Reviewer #2:**

**Comment 1**

- Please explain what method you have used to select your sample? e.g. randomly, convenience???? Please explain how you select the schools.

**Response 1**

Thank you very much. We are sorry for giving incomplete information about the sample recruitment in our initial version of the manuscript. Following the reviewer’s comment, information regarding the recruitment process as well as about the sample selection has been included in the “Study design and sample selection” section. Please, see lines 75 to 90.

**Comment 2**

Table 2. Please include 95% CI. You may exclude the none significant variables out of the table and explain those in the result?

**Response 2**

Thank you for the suggestion. Following the reviewer’s comment, 95% Confidence Intervals have been included in Table 2. Regarding the suggestion of excluding the non-significant associations out of the table and explaining them in the results section, although it is a good idea, it is not possible due to the PLoS ONE’s data sharing requirements, which “does not permit references to inaccessible data”. In this context, PLoS ONE “requires that authors provide all relevant data within the paper”. Thus, for a better presentation of these results, data in Table 2 has been reorganized into two tables. Please, see Tables 2 and 3.

**Comment 3**

Table 3. could you present table 3 in figure? may be the significant ones?

**Response 3**

Authors thank the reviewer very much for the idea. However, this would cause that part of the results would not be shown, breaching PLoS ONE’s data sharing requirements.

In this line, as requested by the editor, authors have removed the phrase “data not shown” from the results section, which referred to the results from the receiver operating characteristic curve analysis for the diagnostic performance of circulating inflammatory biomarkers in identifying low academic abilities. These data have been now included as a S1 Table (supporting information file). Please, see lines 234 to 235.

**Comment 4**

Minor comment: discussion - lines 211 - You may want to say the main finding of the present study reveals an association between TNF - alpha and math........ please fix similar wordings throughout.

**Response 4**

We would like to thank the reviewer for the appreciation and apologize for the inconsistency. We have modified the terms used to be consistent throughout the manuscript. However, we have included the term "with" twice for a better understanding. Please, see lines 255 to 257, 274 to 276, 288 to 289, and 335 to 336.

**Comment 5**

Major comment.

We know that cognition as well as academic performance are results of various factors including nutrition, socio-economic factors, health status, genetic, environment etc or we can say long term effect of various factors. We also know that inflammation markers such as CRP and TNF alpha are early (maximum of 48 hrs) markers of inflammation or infection.

Now, my question is on the research question - are you trying to answer that inflammation is associated with cognitive and/or academic performance or you are tying to show that inflammation markers are associated with for instance, neurogenesis??? you have provided one in vitro-study that shows this relationship, is there any evidence from in vivo study that you could provide?

simple example. If the subjects were free of inflammation three days or more before data collection would the result be different?

**Response 5**

Thank you very much for the appreciation. In the present study, authors aimed to examine the association between inflammatory biomarkers and academic performance. Thus, with the intention to avoid confusion, that information from an in vitro study has been clarified, and results from studies in humans have been also included to support the possible reasons underlying the associations found. Please, see lines 277 to 287.

Participants self-reported whether they had experienced symptoms of any general health problem (e.g., cold, infection, fever, allergies) or dental problems (e.g., toothache) during the past week, as well as at the time of testing. Thus, participants who reported some of these health problems were given an appointment some weeks later when they self-reported not to be experiencing health problems, and we assumed that adolescents recruited for the present study were apparently healthy. This important point has been included in the methods section, and since health status was not assessed by a physician, this issue has been commented in the limitations section. Please, see lines 90 to 93, and 317 to 322.

Lastly, according to the reviewer’s comment, since inflammatory levels were not measured several times and health status was not checked by a physician, these issues, which could influence the study associations, have been included as weakness of our study in the limitations and strengths section. Please, see lines 322 to 323.

**Comment 6**

Conclusion: should be based on your data.

**Response 6**

Thank you very much for the comment. Following the reviewer’s request, the conclusions section has been rewritten to capture the results of the current study. Please, see lines 37 to 40, and 332 to 341.
